# COVID-19: Emergence, Spread, Possible Treatments, and Global Burden

**DOI:** 10.3389/fpubh.2020.00216

**Published:** 2020-05-28

**Authors:** Raghuvir Keni, Anila Alexander, Pawan Ganesh Nayak, Jayesh Mudgal, Krishnadas Nandakumar

**Affiliations:** ^1^Department of Pharmacology, Manipal College of Pharmaceutical Sciences, Manipal Academy of Higher Education, Manipal, India; ^2^Department of Health Sciences, School of Education and Health, Cape Breton University, Sydney, NS, Canada

**Keywords:** 2019-nCoV, COVID-19, SARS-CoV-2, coronavirus, pandemic, SARS

## Abstract

The Coronavirus (CoV) is a large family of viruses known to cause illnesses ranging from the common cold to acute respiratory tract infection. The severity of the infection may be visible as pneumonia, acute respiratory syndrome, and even death. Until the outbreak of SARS, this group of viruses was greatly overlooked. However, since the SARS and MERS outbreaks, these viruses have been studied in greater detail, propelling the vaccine research. On December 31, 2019, mysterious cases of pneumonia were detected in the city of Wuhan in China's Hubei Province. On January 7, 2020, the causative agent was identified as a new coronavirus (2019-nCoV), and the disease was later named as COVID-19 by the WHO. The virus spread extensively in the Wuhan region of China and has gained entry to over 210 countries and territories. Though experts suspected that the virus is transmitted from animals to humans, there are mixed reports on the origin of the virus. There are no treatment options available for the virus as such, limited to the use of anti-HIV drugs and/or other antivirals such as Remdesivir and Galidesivir. For the containment of the virus, it is recommended to quarantine the infected and to follow good hygiene practices. The virus has had a significant socio-economic impact globally. Economically, China is likely to experience a greater setback than other countries from the pandemic due to added trade war pressure, which have been discussed in this paper.

## Introduction

*Coronaviridae* is a family of viruses with a positive-sense RNA that possess an outer viral coat. When looked at with the help of an electron microscope, there appears to be a unique corona around it. This family of viruses mainly cause respiratory diseases in humans, in the forms of common cold or pneumonia as well as respiratory infections. These viruses can infect animals as well (1, 2). Up until the year 2003, coronavirus (CoV) had attracted limited interest from researchers. However, after the SARS (severe acute respiratory syndrome) outbreak caused by the SARS-CoV, the coronavirus was looked at with renewed interest (3, 4). This also happened to be the first epidemic of the 21st century originating in the Guangdong province of China. Almost 10 years later, there was a MERS (Middle East respiratory syndrome) outbreak in 2012, which was caused by the MERS-CoV (5, 6). Both SARS and MERS have a zoonotic origin and originated from bats. A unique feature of these viruses is the ability to mutate rapidly and adapt to a new host. The zoonotic origin of these viruses allows them to jump from host to host. Coronaviruses are known to use the angiotensin-converting enzyme-2 (ACE-2) receptor or the dipeptidyl peptidase IV (DPP-4) protein to gain entry into cells for replication (7–10).

In December 2019, almost seven years after the MERS 2012 outbreak, a novel Coronavirus (2019-nCoV) surfaced in Wuhan in the Hubei region of China. The outbreak rapidly grew and spread to neighboring countries. However, rapid communication of information and the increasing scale of events led to quick quarantine and screening of travelers, thus containing the spread of the infection. The major part of the infection was restricted to China, and a second cluster was found on a cruise ship called the Diamond Princess docked in Japan (11, 12).

## Origin

The new virus was identified to be a novel Coronavirus and was thus initially named 2019-nCoV; later, it was renamed severe acute respiratory syndrome coronavirus 2 (SARS-CoV-2) (13), and the disease it causes is now referred to as Coronavirus Disease-2019 (COVID-19) by the WHO. The virus was suspected to have begun its spread in the Huanan seafood wholesale market in the Wuhan region. It is possible that an animal that was carrying the virus was brought into or sold in the market, causing the spread of the virus in the crowded marketplace. One of the first claims made was in an article published in the Journal of Medical Virology (14), which identified snakes as the possible host. A second possibility was that pangolins could be the wild host of SARS-CoV-2 (15), though the most likely possibility is that the virus originated from bats (13, 16–19). Increasing evidence and experts are now collectively concluding the virus had a natural origin in bats, as with previous such respiratory viruses (2, 20–24).

Similarly, SARS and MERS were also suspected to originate from bats. In the case of MERS, the dromedary camel is an intermediate host (5, 10). Bats have been known to harbor coronaviruses for quite some time now. Just as in the case of avian flu, SARS, MERS, and possibly even HIV, with increasing selection and ecological pressure due to human activities, the virus made the jump from animal to man. Humans have been encroaching increasingly into forests, and this is true over much of China, as in Africa. Combined with additional ecological pressure due to climate change, such zoonotic spillovers are now more common than ever. It is likely that the next disease X will also have such an origin (25). We have learned the importance of identification of the source organism due to the Ebola virus pandemic. Viruses are unstable organisms genetically, constantly mutating by genetic shift or drift. It is not possible to predict when a cross-species jump may occur and when a seemingly harmless variant form of the virus may turn into a deadly strain. Such an incident occurred in Reston, USA, with the Reston virus (26), an alarming reminder of this possibility. The identification of the original host helps us to contain future spreads as well as to learn about the mechanism of transmission of viruses. Until the virus is isolated from a wild animal host, in this case, mostly bats, the zoonotic origin will remain hypothetical, though likely. It should further be noted that the virus has acquired several mutations, as noted by a group in China, indicating that there are more than two strains of the virus, which may have had an impact on its pathogenicity. However, this claim remains unproven, and many experts have argued otherwise; data proving this are not yet available (27). A similar finding was reported from Italy and India independently, where they found two strains (28, 29). These findings need to be further cross-verified by similar analyses globally. If true, this finding could effectively explain why some nations are more affected than others.

## Transmission

**Figure 1:**
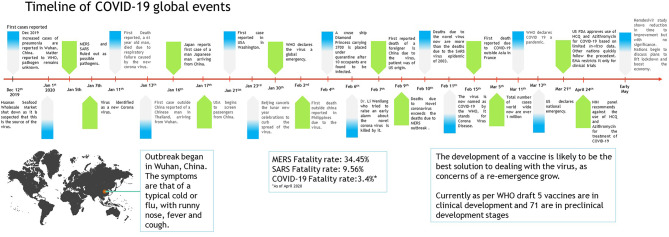
Timeline of COVID-19 progression (30–32).

**Figure 2:**
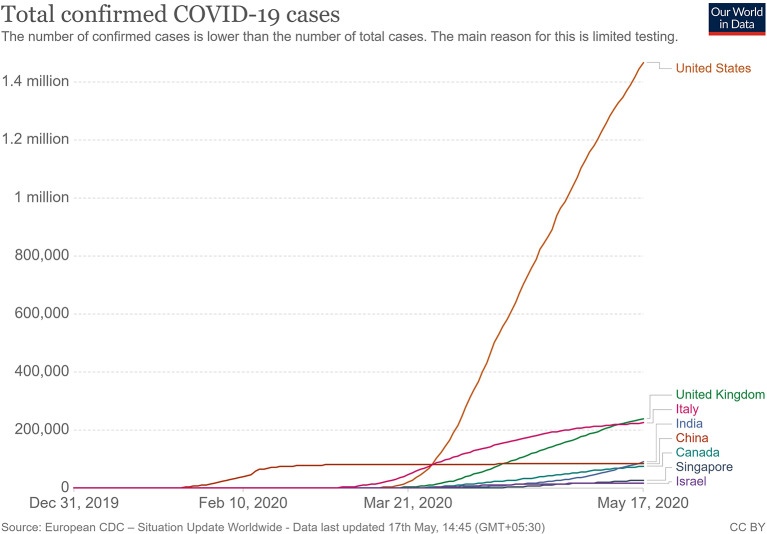
Total confirmed COVID 19 cases as of May 2020 (33).

When the spread of COVID-19 began (Figure 1), the virus appeared to be contained within China and the cruise ship “Diamond Princess,” which formed the major clusters of the virus. However, as of April 2020, over 210 countries and territories are affected by the virus, with Europe, the USA, and Iran forming the new cluster of the virus. The USA (Figure 2) has the highest number of confirmed COVID-19 cases, whereas India and China, despite being among the most population-dense countries in the world, have managed to constrain the infection rate by the implementation of a complete lockdown with arrangements in place to manage the confirmed cases. Similarly, the UK has also managed to maintain a low curve of the graph by implementing similar measures, though it was not strictly enforced. Reports have indicated that the presence of different strains or strands of the virus may have had an effect on the management of the infection rate of the virus (27–29). The disease is spread by droplet transmission. As of April 2020, the total number of infected individuals stands at around 3 million, with ~200,000 deaths and more than 1 million recoveries globally (30, 34). The virus thus has a fatality rate of around 2% and an R_0_ of 3 based on current data. However, a more recent report from the CDC, Atlanta, USA, claims that the R_0_ could be as high as 5.7 (35). It has also been observed from data available from China and India that individuals likely to be infected by the virus from both these countries belong to the age groups of 20–50 years (36, 37). In both of these countries, the working class mostly belongs to this age group, making exposure more likely. Germany and Singapore are great examples of countries with a high number of cases but low fatalities as compared to their immediate neighbors. Singapore is one of the few countries that had developed a detailed plan of action after the previous SARS outbreak to deal with a similar situation in the future, and this worked in their favor during this outbreak. Both countries took swift action after the outbreak began, with Singapore banning Chinese travelers and implementing screening and quarantine measures at a time when the WHO recommended none. They ordered the elderly and the vulnerable to strictly stay at home, and they ensured that lifesaving equipment and large-scale testing facilities were available immediately (38, 39). Germany took similar measures by ramping up testing capacity quite early and by ensuring that all individuals had equal opportunity to get tested. This meant that young, old, and at-risk people all got tested, thus ensuring positive results early during disease progression and that most cases were mild like in Singapore, thus maintaining a lower death percentage (40). It allowed infected individuals to be identified and quarantined before they even had symptoms. Testing was carried out at multiple labs, reducing the load and providing massive scale, something which countries such as the USA did quite late and India restricted to select government and private labs. The German government also banned large gatherings and advocated social distancing to further reduce the spread, though unlike India and the USA, this was done quite late. South Korea is another example of how a nation has managed to contain the spread and transmission of the infection. South Korea and the USA both reported their first COVID-19 cases on the same day; however, the US administration downplayed the risks of the disease, unlike South Korean officials, who constantly informed their citizens about the developments of the disease using the media and a centralized messaging system. They also employed the Trace, Test, and Treat protocol to identify and isolate patients fast, whereas the USA restricted this to patients with severe infection and only later broadened this criterion, like many European countries as well as India. Unlike the USA, South Korea also has universal healthcare, ensuring free diagnostic testing.

The main mode of transmission of 2019-nCoV is human to human. As of now, animal-to-human transfer has not yet been confirmed. Asymptomatic carriers of the virus are at major risk of being superinfectors with this disease, as all those infected may not develop the disease (41). This is a concern that has been raised by nations globally, with the Indian government raising concerns on how to identify and contain asymptomatic carriers, who could account for 80% of those infected (42). Since current resources are directed towards understanding the hospitalized individuals showing symptoms, there is still a vast amount of information about asymptomatic individuals that has yet to be studied. For example, some questions that need to be answered include: Do asymptomatic individuals develop the disease at any point in time at all? Do they eventually develop antibodies? How long do they shed the virus for? Can any tissue of these individuals store the virus in a dormant state? Asymptomatic transmission is a gray area that encompasses major unknowns in COVID-19.

The main route of human-to-human transmission is by droplets, which are generated during coughing, talking, or sneezing and are then inhaled by a healthy individual. They can also be indirectly transmitted to a person when they land on surfaces that are touched by a healthy individual who may then touch their nose, mouth, or eyes, allowing the virus entry into the body. Fomites are also a common issue in such diseases (43).

Aerosol-based transmission of the virus has not yet been confirmed (43). Stool-based transmission via the fecal-oral route may also be possible since the SARS-CoV-2 has been found in patient feces (44, 45). Some patients with COVID-19 tend to develop diarrhea, which can become a major route of transmission if proper sanitation and personal hygiene needs are not met. There is no evidence currently available to suggest intrauterine vertical transmission of the disease in pregnant women (46).

More investigation is necessary of whether climate has played any role in the containment of the infection in countries such as India, Singapore, China, and Israel, as these are significantly warmer countries as compared with the UK, the USA, and Canada (Figure 2). Ideally, a warm climate should prevent the virus from surviving for longer periods of time on surfaces, reducing transmissibility.

## Pathophysiology

On gaining entry via any of the mucus membranes, the single-stranded RNA-based virus enters the host cell using type 2 transmembrane serine protease (TMPRSS2) and ACE2 receptor protein, leading to fusion and endocytosis with the host cell (47–49). The uncoated RNA is then translated, and viral proteins are synthesized. With the help of RNA-dependant RNA polymerase, new RNA is produced for the new virions. The cell then undergoes lysis, releasing a load of new virions into the patients' body. The resultant infection causes a massive release of pro-inflammatory cytokines that causes a cytokine storm.

## Clinical Presentation

**Figure 3:**
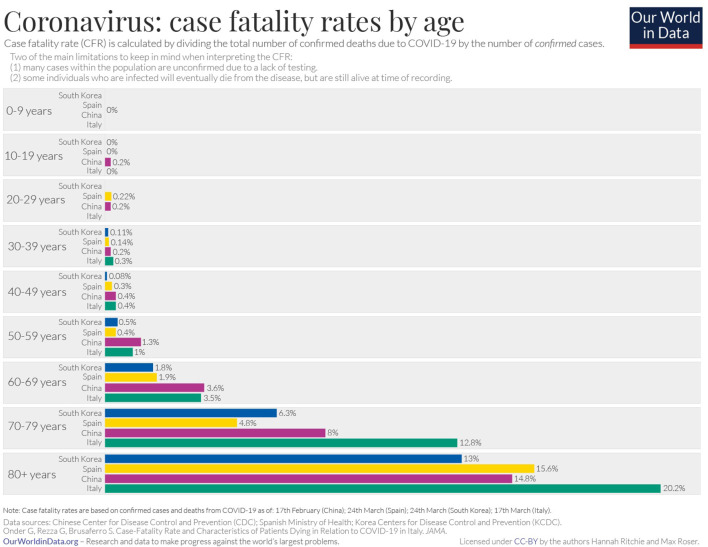
Case fatality rate by age in selected countries as of April 2020 (33).

**Figure 4:**
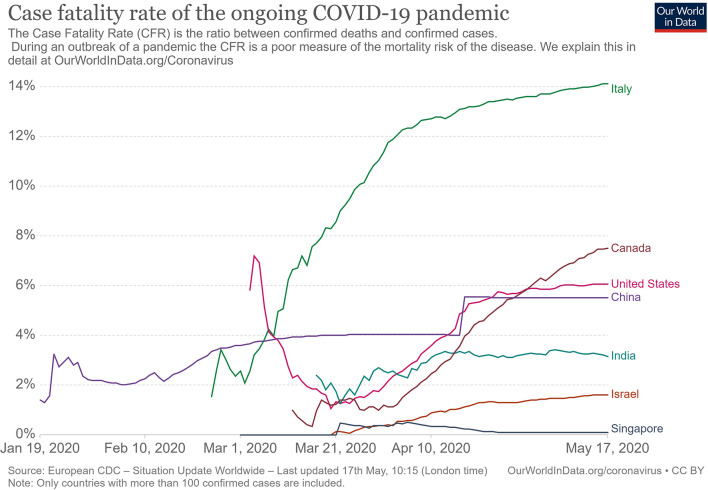
Case fatality rate in selected countries (33).

**Figure 5:**
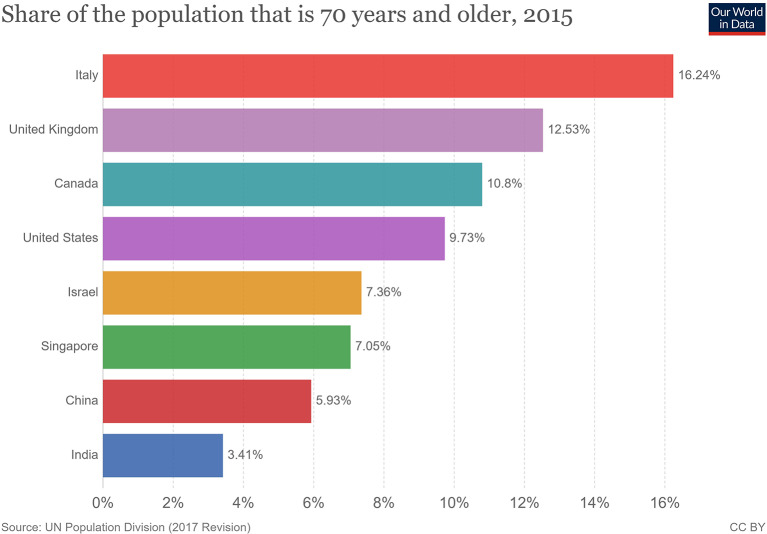
Population share above 70 years of age (33).

The clinical presentation of the disease resembles beta coronavirus infections. The virus has an incubation time of 2–14 days, which is the reason why most patients suspected to have the illness or contact with an individual having the illness remain in quarantine for the said amount of time. Infection with SARS-CoV-2 causes severe pneumonia, intermittent fever, and cough (50, 51). Symptoms of rhinorrhoea, pharyngitis, and sneezing have been less commonly seen. Patients often develop acute respiratory distress syndrome within 2 days of hospital admission, requiring ventilatory support. It has been observed that during this phase, the mortality tends to be high. Chest CT will show indicators of pneumonia and ground-glass opacity, a feature that has helped to improve the preliminary diagnosis (51). The primary method of diagnosis for SARS-CoV-2 is with the help of PCR. For the PCR testing, the US CDC recommends testing for the N gene, whereas the Chinese CDC recommends the use of ORF lab and N gene of the viral genome for testing. Some also rely on the radiological findings for preliminary screening (52). Additionally, immunodiagnostic tests based on the presence of antibodies can also play a role in testing. While the WHO recommends the use of these tests for research use, many countries have pre-emptively deployed the use of these tests in the hope of ramping up the rate and speed of testing (52–54). Later, they noticed variations among the results, causing them to stop the use of such kits; there was also debate among the experts about the sensitivity and specificity of the tests. For immunological tests, it is beneficial to test for antibodies against the virus produced by the body rather than to test for the presence of the viral proteins, since the antibodies can be present in larger titers for a longer span of time. However, the cross-reactivity of these tests with other coronavirus antibodies is something that needs verification. Biochemical parameters such as D-dimer, C-reactive protein, and variations in neutrophil and lymphocyte counts are some other parameters that can be used to make a preliminary diagnosis; however, these parameters vary in a number of diseases and thus cannot be relied upon conclusively (51). Patients with pre-existing diseases such as asthma or similar lung disorder are at higher risk, requiring life support, as are those with other diseases such as diabetes, hypertension, or obesity. Those above the age of 60 have displayed the highest mortality rate in China, a finding that is mirrored in other nations as well (Figure 3) (55). If we cross-verify these findings with the population share that is above the age of 70, we find that Italy, the United Kingdom, Canada, and the USA have one of the highest elderly populations as compared to countries such as India and China (Figure 4), and this also reflects the case fatality rates accordingly (Figure 5) (33). This is a clear indicator that aside from comorbidities, age is also an independent risk factor for death in those infected by COVID-19. Also, in the US, it was seen that the rates of African American deaths were higher. This is probably due to the fact that the prevalence of hypertension and obesity in this community is higher than in Caucasians (56, 57). In late April 2020, there are also claims in the US media that young patients in the US with COVID-19 may be at increased risk of stroke; however, this is yet to be proven. We know that coagulopathy is a feature of COVID-19, and thus stroke is likely in this condition (58, 59). The main cause of death in COVID-19 patients was acute respiratory distress due to the inflammation in the linings of the lungs caused by the cytokine storm, which is seen in all non-survival cases and in respiratory failure. The resultant inflammation in the lungs, served as an entry point of further infection, associated with coagulopathy end-organ failure, septic shock, and secondary infections leading to death (60–63).

## Treatment

For COVID-19, there is no specific treatment available. The WHO announced the organization of a trial dubbed the “Solidarity” clinical trial for COVID-19 treatments (64). This is an international collaborative study that investigates the use of a few prime candidate drugs for use against COVID-19, which are discussed below. The study is designed to reduce the time taken for an RCT by over 80%. There are over 1087 studies (Supplementary Data 1) for COVID-19 registered at clinicaltrials.gov, of which 657 are interventional studies (Supplementary Data 2) (65). The primary focus of the interventional studies for COVID-19 has been on antimalarial drugs and antiviral agents (Table 1), while over 200 studies deal with the use of different forms of oxygen therapy. Most trials focus on improvement of clinical status, reduction of viral load, time to improvement, and reduction of mortality rates. These studies cover both severe and mild cases.

**Table 1:** List of therapeutic drugs under study for COVID-19 as per clinical trials registered under clinicaltrials.gov.

**Drug class**	**Drug list**
Antiviral	Remdesivir, Lopinavir/ritonavir, Favipiravir, Oseltamivir
Antiprotozoal drugs	Hydroxychloroquine, Chloroquine, Nitazoxanide
Vaccines, immunoglobulin therapies, and immunostimulants	Convalescent plasma, BCG Vaccine, Levamisole/Isoprinosine
Biologicals and kinase inhibitors	Tocilizumab, Canakinumab, Siltuximab, Lenzilumab, Ravulizumab, Sarilumab, Imatinib, Baricitinib, Ruxolitinib
Antibacterial drugs	Azithromycin, Amoxicillin/Clavulanate
Cardiovascular drugs	Telmisartan, Sildenafil citrate, Losartan, Simvastatin, Enoxaparin, Aspirin, Nafamostat mesilate
Other agents	Naproxen
	Colchicine
	Isotretinoin
	Levamisole, Ivermectin
	Almitrine
	Anakinra
	Tacrolimus
	Deferoxamine
	Famotidine
Non-pharmacological interventions	Diet, Non-invasive oxygenation, Intubation, Hyperbaric oxygenation

### Use of Antimalarial Drugs Against SARS-CoV-2

The use of chloroquine for the treatment of corona virus-based infection has shown some benefit in the prevention of viral replication in the cases of SARS and MERS. However, it was not validated on a large scale in the form of a randomized control trial (50, 66–68). The drugs of choice among antimalarials are Chloroquine (CQ) and Hydroxychloroquine (HCQ). The use of CQ for COVID-19 was brought to light by the Chinese, especially by the publication of a letter to the editor of Bioscience Trends by Gao et al. (69). The letter claimed that several studies found CQ to be effective against COVID-19; however, the letter did not provide many details. Immediately, over a short span of time, interest in these two agents grew globally. Early *in vitro* data have revealed that chloroquine can inhibit the viral replication (70, 71).

HCQ and CQ work by raising the pH of the lysosome, the cellular organelle that is responsible for phagocytic degradation. Its function is to combine with cell contents that have been phagocytosed and break them down eventually, in some immune cells, as a downstream process to display some of the broken proteins as antigens, thus further enhancing the immune recruitment against an antigen/pathogen. The drug was to be administered alone or with azithromycin. The use of azithromycin may be advocated by the fact that it has been seen previously to have some immunomodulatory role in airway-related disease. It appears to reduce the release of pro-inflammatory cytokines in respiratory illnesses (72). However, HCQ and azithromycin are known to have a major drug interaction when co-administered, which increases the risk of QT interval prolongation (73). Quinine-based drugs are known to have adverse effects such as QT prolongation, retinal damage, hypoglycemia, and hemolysis of blood in patients with G-6-PD deficiency (66). Several preprints, including, a metanalysis now indicate that HCQ may have no benefit for severe or critically ill patients who have COVID-19 where the outcome is need for ventilation or death (74, 75). As of April 21, 2020, after having pre-emptively recommended their use for SARS-CoV-2 infection, the US now advocates against the use of these two drugs based on the new data that has become available.

### Use of Antiviral Drugs Against SARS-CoV-2

The antiviral agents are mainly those used in the case of HIV/AIDS, these being Lopinavir and Ritonavir. Other agents such as nucleoside analogs like Favipiravir, Ribavirin, Remdesivir, and Galidesivir have been tested for possible activity in the prevention of viral RNA synthesis (76). Among these drugs, Lopinavir, Ritonavir, and Remdesivir are listed in the Solidarity trial by the WHO.

Remdesivir is a nucleotide analog for adenosine that gets incorporated into the viral RNA, hindering its replication and causing chain termination. This agent was originally developed for Ebola Virus Disease (77). A study was conducted with rhesus macaques infected with SARS-CoV-2 (78). In that study, after 12 h of infection, the monkeys were treated with either Remdesivir or vehicle. The drug showed good distribution in the lungs, and the animals treated with the drug showed a better clinical score than the vehicle group. The radiological findings of the study also indicated that the animals treated with Remdesivir have less lung damage. There was a reduction in viral replication but not in virus shedding. Furthermore, there were no mutations found in the RNA polymerase sequences. A randomized clinical control study that became available in late April 2020 (79), having 158 on the Remdesivir arm and 79 on the placebo arm, found that Remdesivir reduced the time to recovery in the Remdesivir-treated arm to 11 days, while the placebo-arm recovery time was 15 days. Though this was not found to be statistically significant, the agent provided a basis for further studies. The 28-days mortality was found to be similar for both groups. This has now provided us with a basis on which to develop future molecules. The study has been supported by the National Institute of Health, USA. The authors of the study advocated for more clinical trials with Remdesivir with a larger population. Such larger studies are already in progress, and their results are awaited. Remdesivir is currently one of the drugs that hold most promise against COVID-19.

An early trial in China with Lopinavir and Ritonavir showed no benefit compared with standard clinical care (80). More studies with this drug are currently underway, including one in India (81, 82).

### Use of Convalescent Patient Plasma

Another possible option would be the use of serum from convalescent individuals, as this is known to contain antibodies that can neutralize the virus and aid in its elimination. This has been tried previously for other coronavirus infections (83). Early emerging case reports in this aspect look promising compared to other therapies that have been tried (84–87). A report from China indicates that five patients treated with plasma recovered and were eventually weaned off ventilators (84). They exhibited reductions in fever and viral load and improved oxygenation. The virus was not detected in the patients after 12 days of plasma transfusion. The US FDA has provided detailed recommendations for investigational COVID-19 Convalescent Plasma use (88). One of the benefits of this approach is that it can also be used for post-exposure prophylaxis. This approach is now beginning to be increasingly adopted in other countries, with over 95 trials registered on clinicaltrials.gov alone, of which at least 75 are interventional (89). The use of convalescent patient plasma, though mostly for research purposes, appears to be the best and, so far, the only successful option for treatment available.

From a future perspective, the use of monoclonal antibodies for the inhibition of the attachment of the virus to the ACE-2 receptor may be the best bet. Aside from this, ACE-2-like molecules could also be utilized to attach and inactivate the viral proteins, since inhibition of the ACE-2 receptor would not be advisable due to its negative repercussions physiologically. In the absence of drug regimens and a vaccine, the treatment is symptomatic and involves the use of non-invasive ventilation or intubation where necessary for respiratory failure patients. Patients that may go into septic shock should be managed as per existing guidelines with hemodynamic support as well as antibiotics where necessary.

## Prevention

The WHO has recommended that simple personal hygiene practices can be sufficient for the prevention of spread and containment of the disease (90). Practices such as frequent washing of soiled hands or the use of sanitizer for unsoiled hands help reduce transmission. Covering of mouth while sneezing and coughing, and disinfection of surfaces that are frequently touched, such as tabletops, doorknobs, and switches with 70% isopropyl alcohol or other disinfectants are broadly recommended. It is recommended that all individuals afflicted by the disease, as well as those caring for the infected, wear a mask to avoid transmission. Healthcare works are advised to wear a complete set of personal protective equipment as per WHO-provided guidelines. Fumigation of dormitories, quarantine rooms, and washing of clothes and other fomites with detergent and warm water can help get rid of the virus. Parcels and goods are not known to transmit the virus, as per information provided by the WHO, since the virus is not able to survive sufficiently in an open, exposed environment. Quarantine of infected individuals and those who have come into contact with an infected individual is necessary to further prevent transmission of the virus (91). Quarantine is an age-old archaic practice that continues to hold relevance even today for disease containment. With the quarantine being implemented on such a large scale in some countries, taking the form of a national lockdown, the question arises of its impact on the mental health of all individuals. This topic needs to be addressed, especially in countries such as India and China, where it is still a matter of partial taboo to talk about it openly within the society.

In India, the Ministry of Ayurveda, Yoga, and Naturopathy, Unani, Siddha and Homeopathy (AYUSH), which deals with the alternative forms of medicine, issued a press release that the homeopathic, drug Arsenicum album 30, can be taken on an empty stomach for 3 days to provide protection against the infection (92). It also provided a list of herbal drugs in the same press release as per Ayurvedic and Unani systems of medicine that can boost the immune system to deal with the virus. However, there is currently no evidence to support the use of these systems of medicine against COVID-19, and they need to be tested.

The prevention of the disease with the use of a vaccine would provide a more viable solution. There are no vaccines available for any of the coronaviruses, which includes SARS and MERS. The development of a vaccine, however, is in progress at a rapid pace, though it could take about a year or two. As of April 2020, no vaccine has completed the development and testing process. A popular approach has been with the use of mRNA-based vaccine (93–96). mRNA vaccines have the advantage over conventional vaccines in terms of production, since they can be manufactured easily and do not have to be cultured, as a virus would need to be. Alternative conventional approaches to making a vaccine against SARS-CoV-2 would include the use of live attenuated virus as well as using the isolated spike proteins of the virus. Both of these approaches are in progress for vaccine development (97). Governments across the world have poured in resources and made changes in their legislation to ensure rapid development, testing, and deployment of a vaccine.

## Barriers to Treatment

### Lack of Transparency and Poor Media Relations

The lack of government transparency and poor reporting by the media have hampered the measures that could have been taken by healthcare systems globally to deal with the COVID-19 threat. The CDC, as well as the US administration, downplayed the threat and thus failed to stock up on essential supplies, ventilators, and test kits. An early warning system, if implemented, would have caused borders to be shut and early lockdowns. The WHO also delayed its response in sounding the alarm regarding the severity of the outbreak to allow nations globally to prepare for a pandemic. Singapore is a prime example where, despite the WHO not raising concerns and banning travel to and from China, a country banned travelers and took early measures, thus managing the outbreak quite well. South Korea is another example of how things may have played out had those measures by agencies been taken with transparency. Increased transparency would have allowed the healthcare sector to better prepare and reduced the load of patients they had to deal with, helping flatten the curve. The increased patient load and confusion among citizens arising from not following these practices has proved to be a barrier to providing effective treatments to patients with the disease elsewhere in the world.

### Lack of Preparedness and Protocols

Despite the previous SARS outbreak teaching us important lessons and providing us with data on a potential outbreak, many nations did not take the important measures needed for a future outbreak. There was no allocation of sufficient funds for such an event. Many countries experienced severe lack of PPE, and the lockdown precautions hampered the logistics of supply and manufacturing of such essential equipment. Singapore and South Korea had protocols in place and were able to implement them at a moment's notice. The spurt of cases that Korea experienced was managed well, providing evidence to this effect. The lack of preparedness and lack of protocol in other nations has resulted in confusion as to how the treatment may be administered safely to the large volume of patients while dealing with diagnostics. Both of these factors have limited the accessibility to healthcare services due to sheer volume.

## Socio-Economic Impact

During the SARS epidemic, China faced an economic setback, and experts were unsure if any recovery would be made. However, the global and domestic situation was then in China's favor, as it had a lower debt, allowing it to make a speedy recovery. This is not the case now. Global experts have a pessimistic outlook on the outcome of this outbreak (98). The fear of COVID-19 disease, lack of proper understanding of the dangers of the virus, and the misinformation spread on the social media (99) have caused a breakdown of the economic flow globally (100). An example of this is Indonesia, where a great amount of fear was expressed in responses to a survey when the nation was still free of COVID-19 (101). The pandemic has resulted in over 2.6 billion people being put under lockdown. This lockdown and the cancellation of the lunar year celebration has affected business at the local level. Hundreds of flights have been canceled, and tourism globally has been affected. Japan and Indonesia are estimated to lose over 2.44 billion dollars due to this (102, 103). Workers are not able to work in factories, transportation in all forms is restricted, and goods are not produced or moved. The transport of finished products and raw materials out of China is low. The Economist has published US stock market details indicating that companies in the US that have Chinese roots fell, on average, 5 points on the stock market as compared to the S&P 500 index (104). Companies such as Starbucks have had to close over 4,000 outlets due to the outbreak as a precaution. Tech and pharma companies are at higher risk since they rely on China for the supply of raw materials and active pharmaceutical ingredients. Paracetamol, for one, has reported a price increase of over 40% in India (104–106). Mass hysteria in the market has caused selling of shares of these companies, causing a tumble in the Indian stock market. Though long-term investors will not be significantly affected, short-term traders will find themselves in soup. Politically, however, this has further bolstered support for world leaders in countries such as India, Germany, and the UK, who are achieving good approval ratings, with citizens being satisfied with the government's approach. In contrast, the ratings of US President Donald Trump have dropped due to the manner in which the COVID-19 pandemic was handled. These minor impacts may be of temporary significance, and the worst and direct impact will be on China itself (107–109), as the looming trade war with the USA had a negative impact on the Chinese and Asian markets. The longer production of goods continues to remain suspended, the more adversely it will affect the Chinese economy and the global markets dependent on it (110). If this disease is not contained, more and more lockdowns by multiple nations will severely affect the economy and lead to many social complications.

## Conclusion

The appearance of the 2019 Novel Coronavirus has added and will continue to add to our understanding of viruses. The pandemic has once again tested the world's preparedness for dealing with such outbreaks. It has provided an outlook on how a massive-scale biological event can cause a socio-economic disturbance through misinformation and social media. In the coming months and years, we can expect to gain further insights into SARS-CoV-2 and COVID-19.

## Author Contributions

KN: conceptualization. RK, AA, JM, and KN: investigation. RK and AA: writing—original draft preparation. KN, PN, and JM: writing—review and editing. KN: supervision.

## Conflict of Interest

The authors declare that the research was conducted in the absence of any commercial or financial relationships that could be construed as a potential conflict of interest.

## Supplementary Material

Supplementary Data 1, 2 List of all studies registered for COVID-19 on clinicaltrials.gov.
